# Draft Genome Sequences of *Salmonella enterica* subsp. *enterica* Serovar Typhimurium Strains Isolated from Chicken and Swine Carcasses in Two Distinct Geographical Regions from Rio de Janeiro State, Brazil

**DOI:** 10.1128/genomeA.00197-17

**Published:** 2017-04-27

**Authors:** Pedro Henrique N. Panzenhagen, Claudius C. Cabral, Philip N. Suffys, Maria Helena C. Aquino, Robson M. Franco, Virgínia L. A. Pereira, Dalia P. Rodrigues, Carlos A. Conte-Junior

**Affiliations:** a Food Science Program, Chemistry Institute, University Federal of Rio de Janeiro (UFRJ), Rio de Janeiro, Brazil; b Department of Food Technology, Faculty of Veterinary Medicine, Federal Fluminense University (UFF), Niterói, Rio de Janeiro, Brazil; c Faculty of Veterinary Medicine, Severino Sombra University (USS), Vassouras, Rio de Janeiro, Brazil; d Laboratory of Molecular Biology and Diagnosis of Infectious Diseases, Department of Biochemistry and Molecular Biology, Oswaldo Cruz Institute, Oswaldo Cruz Foundation (FIOCRUZ), Rio de Janeiro, Brazil; e Department of Veterinary Collective Health and Public Health, Federal Fluminense University (UFF), Niterói, Rio de Janeiro, Brazil; f National Reference Laboratory Diagnosis of Enteric Bacteria, Oswaldo Cruz Institute, Oswaldo Cruz Foundation (FIOCRUZ), Rio de Janeiro, Brazil; g National Institute of Quality Control in Health, Oswaldo Cruz Foundation (FIOCRUZ), Rio de Janeiro, Brazil

## Abstract

*Salmonella enterica* subsp. *enterica* serovar Typhimurium is a surveyed worldwide serotype with well-characterized genomes for several different strains. In Brazil, very few studies have submitted whole-genome sequences to GenBank. This genome may be useful to analyze the genetic mechanisms comparable to those of other related studies conducted in Brazil and globally.

## GENOME ANNOUNCEMENT

Illness and death from diseases caused by contaminated food are a constant threat to public health and a significant impediment to socioeconomic development worldwide (1). The most frequent causes of foodborne illness are diarrheal disease agents, especially nontyphoidal *Salmonella* spp., which besides norovirus and *Campylobacter* spp., are also responsible for the majority of deaths due to foodborne disease (2). Certainly, *Salmonella enterica* subsp. *enterica* serovar Typhimurium infections represent the majority of this foodborne disease burden, since it leads the top 15 most common serovars worldwide (3).

Through this report, we announce two draft genome sequences that represent a collection of *Salmonella* Typhimurium strains isolated between 2011 and 2015 in two totally distinct geographical regions in Rio de Janeiro State, Brazil. Strain CCRJ_26 is multidrug resistant (MDR) and was isolated in 2015 from a swine carcass swab. Sequencing generated 176 contigs, a G+C content of 52.0%, and a total size of 4.9 Mbp, as expected for *Salmonella* species (4). Strain PPRJ_27 was isolated in 2011 from a chicken carcass swab. Sequencing generated 128 contigs, a G+C content of 52.2%, and a total size of 4.8 Mbp, also as expected for *Salmonella* species. The whole-genome sequencing was generated using paired-end sequencing (2 × 250 bp) and performed on the Illumina MiSeq platform, according to the manufacturer’s suggested protocols for generation of Nextera-XT libraries (5). DNA samples were multiplexed using Illumina-supplied barcodes, and DNA pools were size selected to be in the range of 600 to 1,000 bp (average peaks, ~800 bp). *De novo* assembly was carried out using Geneious 10.0.7 (Biomatters, Auckland, New Zealand), with Velvet 1.2.10 set at default for all parameters (6).

These data are not the first announcements of *S*. Typhimurium sequences in Brazil. Recently, 40 draft genome sequences from a collection of *S*. Typhimurium strains isolated from humans and food in Brazil were submitted to GenBank (7). However, none of those strains come from Rio de Janeiro State in Brazil. We believe that information about our strains may complement that previous publication and will provide support in future research of *S*. Typhimurium molecular epidemiology. More information about the genome characteristics will be detailed in future publications.

### Accession number(s).

This whole-genome shotgun project has been deposited at DDBJ/ENA/GenBank under the accession numbers MVJI00000000 and MVIX00000000. The versions described in this paper are versions MVJI01000000 and MVIX00000000, respectively.
